# Body Structures and Physical Complaints in Upper Limb Reduction Deficiency: A 24-Year Follow-Up Study

**DOI:** 10.1371/journal.pone.0049727

**Published:** 2012-11-30

**Authors:** Sietke G. Postema, Corry K. van der Sluis, Kristina Waldenlöv, Liselotte M. Norling Hermansson

**Affiliations:** 1 Centre for Rehabilitation Research, Örebro County Council, Örebro, Sweden; 2 Department of Rehabilitation Medicine, University of Groningen, University Medical Center Groningen, Groningen, The Netherlands; 3 Limb Deficiency and Arm Prosthesis Centre, Department of Paediatrics, Örebro University Hospital, Örebro, Sweden; 4 Department of Prosthetics and Orthotics, Örebro County Council, Örebro, Sweden; 5 School of Health and Medical Sciences, Örebro University, Örebro, Sweden; The Hebrew University, Israel

## Abstract

**Objective:**

To describe upper body structures associated with upper limb reduction deficiency and the development of these structures over time, to examine the presence of physical complaints in this population, and to compare body structures and complaints between groups based on prosthesis use.

**Design:**

Prospective cohort study with a follow-up period of 24 years, with matched able-bodied controls.

**Subjects:**

Twenty-eight patients with unilateral below-elbow reduction deficiency fitted with myoelectric prostheses, aged 8–18 years at inclusion.

**Method:**

Measurements of upper arm, trunk and spine were performed and study-specific questionnaires were answered at baseline and follow-up; the Brief Pain Inventory and the Quick Disability of Arm, Shoulder, and Hand questionnaires were answered at follow-up.

**Results:**

Both at baseline and follow-up, within-subjects differences in structures of the arm and trunk were shown in patients but not in controls. Spinal deviations, although small, were greater in patients compared to controls. Self-reported disability was higher in patients compared to controls. Differences in back pain and effect of prostheses use could not be shown.

**Conclusions:**

Patients with unilateral below-elbow reduction deficiency have consistent differences in upper body structures. Deviations of the spine, probably of functional origin, do not progress to clinically relevant scoliosis.

## Introduction

Very little is known about the development of the upper body structures of children with upper limb reduction deficiency (ULRD). Examinations of the spine have previously found significant scoliosis without congenital malformations of the spine in 19–31% of children with ULRD [1], [2]. However, the development of these spinal deviations over time is unclear. Current clinical practice encompasses examination of the spine to detect scoliosis, even though scientific evidence for the necessity of this procedure is lacking. Furthermore, little is known about back pain or other physical complaints that these patients may experience in adulthood. One may expect higher rates of physical complaints in the unaffected hand or arm of a person with ULRD after many years of one-handedness. Earlier studies on physical complaints in individuals with a short arm have reported rates of arm and back complaints of 40–55% among the examined population [3]–[6]. These studies did not focus specifically on patients with ULRD, who differ from those with amputations in that they are more able to use both the affected and unaffected arm to perform daily tasks due to natural adaptation. Because of population aging, more insight into physical complaints in older patients is valuable.

Currently, children with ULRD are fitted with a myoelectric prosthesis at an early age [7], [8]. One of the reasons for this is the hypothesis that the weight and use of a prosthesis prevents physical problems at a later age. The use of a prosthesis may stimulate symmetrical movements and the weight may stimulate the growth of bone and soft tissue. However, no research has been performed to study the relation between myoelectric prosthesis use and the development of body structures and physical complaints in patients with ULRD. Hence, there is a need for further studies on this topic.

Therefore this study aims to i) describe the body structures of the spine, trunk and arms in patients with unilateral ULRD compared to able-bodied controls, ii) describe the development of the structures of arms and trunk over time, iii) examine the presence of physical complaints in patients with ULRD compared to able-bodied controls, and iv) compare body structures and physical complaints between groups based on prosthesis use.

## Methods

### Participants and procedure

#### Patients

In 1987, all children with unilateral ULRD below the elbow aged 8–18 years, who were fitted with a myoelectric prosthesis at the Limb Deficiency and Arm Prostheses Centre in Örebro, Sweden, were invited to participate in the study. A further inclusion criterion was sufficient comprehension of the Swedish language. In 2011, the same patients were invited again to the clinic for follow-up measurements.

#### Controls

In 1987, each patient was matched for age and gender with one control from a local school. In 2011, each patient was matched for age, gender, weight (±10 kg), and height (±10 cm) with two employees of the hospital or acquaintances of the researchers. The exclusion criterion for the controls was presence of unilateral upper limb health problems.

In 1987, data were collected during a regular visit to the hospital; in 2011, patients were called in especially for this study. The data was obtained from physical measurements of the spine, trunk and arms, study-specific and validated questionnaires (Table 1). The 1987 data were collected by an occupational therapist and a physician and the 2011 data by a physical therapist and a researcher. Before joining the study, patients and controls received oral and written information and gave their written informed consent. For patients younger than 15 years, written informed consent was given by the parents. The study was approved by the Regional Ethical Review Board in Örebro, Sweden, in 1987 and Uppsala, Sweden, in 2011.

**Table 1:** Data collections performed in 1987 and 2011.

	Research period 1987		Research period 2011	
	Patients	Controls	Patients	Controls
**Physical measurements**				
Structural deviations of the spine:				
Moiré topography	x	x		
Scoliometry			x	x
Perpendicular line			x	x
Scapular size	x	x	x	x
Thoracic circumference			x	x
Upper arm length			x	x
Arm volume	x	x	x	x
Range of motion (shoulder and elbow)			x	x
Leg length inequality			x	x
**Questionnaires**				
Study-specific questionnaire	x	x	x	x
BPI-SF*			x	x
*Quick*DASH§			x	x

* BPI-SF: Short Form of the Brief Pain Inventory.

§ QuickDASH: Shortened version of the Disabilities of the Arm, Shoulder and Hand.

### Physical measurements

#### Structures of the spine

**Figure 1:**
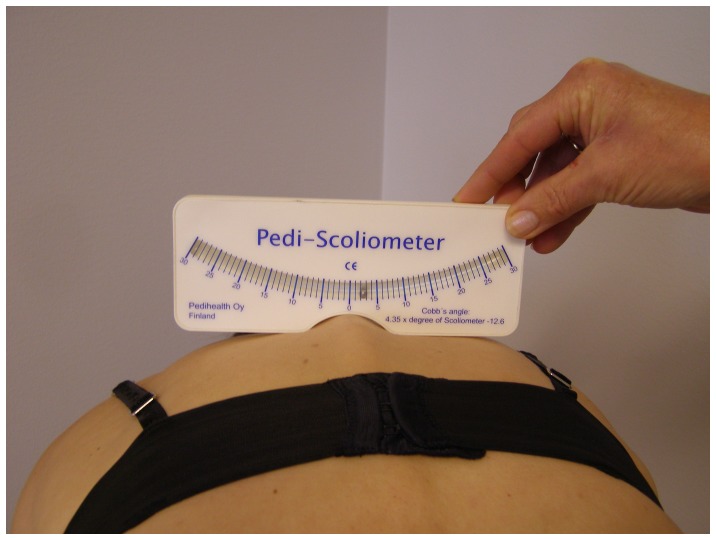
Measurement of deviations of the spine with scoliometry.

In 1987, spinal deviations were measured using Moiré topography [9]. The number of contour lines between the scapula and armpit were recorded for both sides. Measurements were performed with and without prosthesis. There was no correction for leg length inequality (LLI). Due to unavailability of the Moiré topography equipment in 2011, scoliometry was used (Pedi-Scoliometer, Pedihealth Oy, Oulu, Finland). This is a validated method with very good to excellent inter- and intra-rater reliability that shows the angle of trunk rotation (ATR) [10]–[14]. Significant correlations between the Cobb angle and the ATR, especially for the thoracic level, have been shown [15], [16]. Scoliometer measurements were taken from costal level C7 till level L5, by moving down the scoliometer along the spine (Figure 1). The degree and spinal level at the maximal ATR and the side of the corresponding rib hump or lumbar prominence were recorded [2]. In 2011, deviations of the spine were also measured using a perpendicular line. The spinous process of the Th1 was used as the reference point for the vertical line. The level and maximal distance (in 0.5 cm accuracy) of the spinous processes to the string and the side of the deviation was recorded. Both scoliometry and perpendicular line measurements were carried out after correction for LLI and were conducted with and without prosthesis.

#### Structures of the trunk

**Figure 2:**
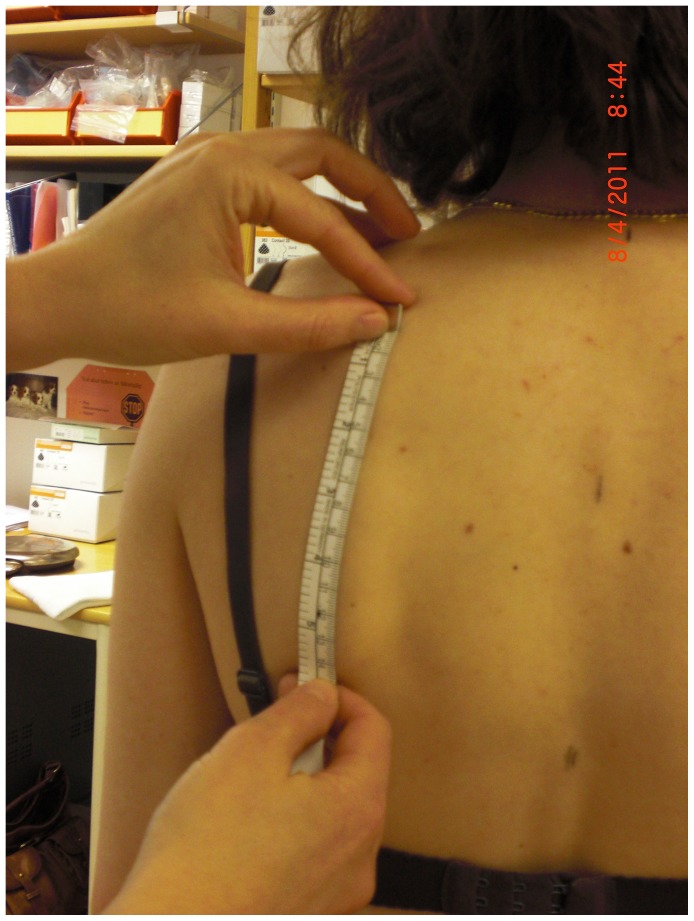
Measurement of scapular size.

**Figure 3:**
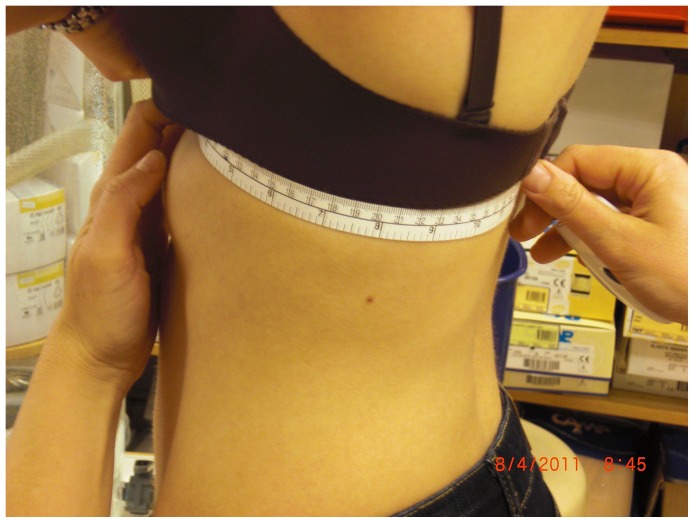
Measurement of thorax circumference.

To measure scapular size and thorax circumference, a measuring tape was used. Scapular size, defined as the distance between the spine of the scapula and the inferior scapular angle alongside the medial border, was measured on both sides (Figure 2). Thorax circumference, defined as the distance between the xiphoid process and the spinous process of Th10, was measured for both sides separately during exhalation (Figure 3).

#### Structures of the arm

**Figure 4:**
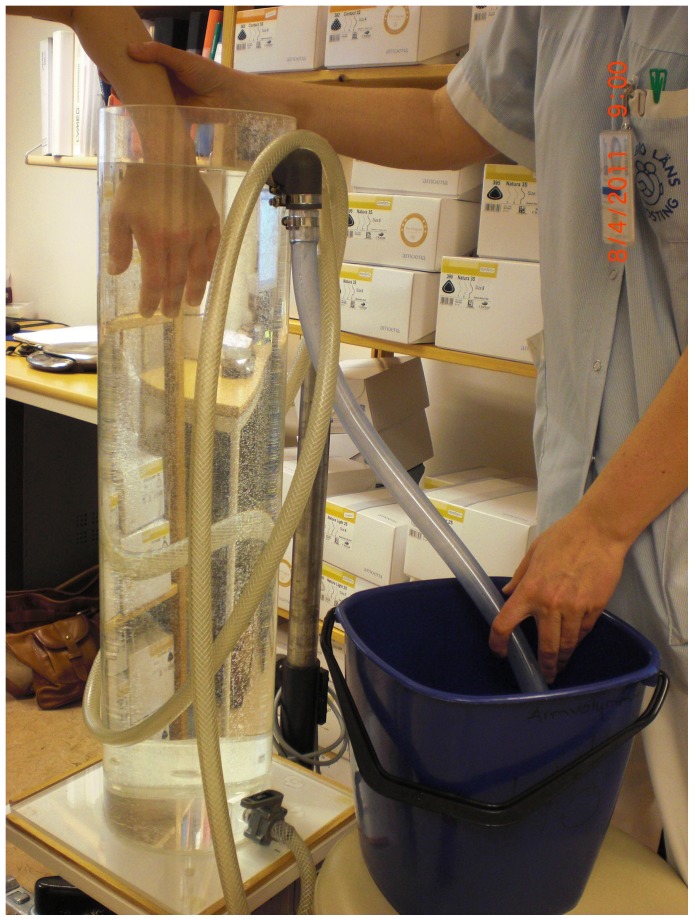
Water plethysmography, example of measuring volume at wrist level.

Water plethysmography was used to measure arm volume in both arms at three predefined points: wrist (level with the styloid process of the radius), elbow (level with the elbow crease, with the elbow flexed at 90°) and upper arm (level with the axilla, with the contralateral arm marked at the same level) (Figure 4). Water plethysmography has a high inter- and intra-rater reliability and is seen as the gold standard for volume measurement of a limb [17]–[19]. The displaced water was measured with 1 ml precision.

Upper arm length was determined by measuring the distance between the lateral side of the acromion and the olecranon.

The range of motion (ROM) of the shoulder was measured for anteflexion, abduction and external rotation, and of the elbow for flexion and extension, using a manual goniometer. Both arms were measured, the deficient side without prosthesis. Differences were calculated as the ROM on the non-deficient side minus the ROM on the deficient side. A difference of 10° or more between either shoulders or elbows was considered to be of clinical relevance [20]. In physical measurements of the able-bodied controls, their non-dominant side was used to correspond to a patient's deficient side.

To measure leg length inequality (LLI), a Pelvic Balance (Otto Bock, Duderstadt, Germany) was used. The endings of the Pelvic Balance were placed on the iliac crests. Differences of ≥0.5 cm were recorded.

### Questionnaires

#### Study specific questionnaires

The 11-item therapist-administered questionnaire used in 1987 recorded age at first fitting, type of prosthesis, frequency of prosthesis use and the presence of back pain. Participants in the control group were only asked about the presence of back pain.

The 15-item self-administered questionnaire used in 2011 contained additional questions about the current work situation and health of the patient. The control group participants answered a 6-item version of this questionnaire.

Frequency of prosthesis use was categorized into two groups: full-time users, defined as wearing the prosthesis at least eight hours a day, seven days a week; and non-full-time users, defined as wearing a prosthesis from never to a maximum of four to eight hours a day, five to seven days a week.

### In 2011 two additional questionnaires were administered

#### Short Form of the Brief Pain Inventory (BPI-SF), Swedish version

The BPI-SF is a validated questionnaire used to assess the intensity of chronic pain, pain relief, pain quality and the interference of the pain on the patient's life [21]–[23]. The first question, concerning pain during the last week, other than common pains like minor headaches, sprains and toothaches, was used to assess the presence of physical complaints.

#### The shortened Disabilities of the Arm, Shoulder and Hand questionnaire (QuickDASH), Swedish version

The *Quick*DASH is a validated 11-item version of the DASH that can be used in upper extremity musculoskeletal disorders with similar precision to the DASH [24], [25]. Answers are ranked on a 5-point Likert scale (‘no pain/difficulties at all’, to ‘a lot of pain/difficulties’). Two additional modules from the DASH, the 4-item sports/music module and the 4-item work module, were also used. The average of the assigned values was transformed to a 0–100 scale score, with a higher score indicating a greater disability.

### Statistical analysis

Statistical analysis was performed using the SPSS 15.0 software package (SPSS Inc., Chicago, USA). Descriptive statistics were calculated for all measurements. The Mann-Whitney U test was used to test for differences between patients and controls and between full-time and non-full-time prosthesis users. The Wilcoxon Signed-Rank test was used to compare the patients' measurements over time and measurements with and without the prosthesis. Fisher's Exact test was used to test for differences in percentages of physical complaints between patients and controls and between full-time prosthesis users and non-full-time users. P-values≤0.05 were considered to indicate statistically significant differences. Data are presented as mean±SD, unless otherwise stated.

## Results

### Demographics

In 1987, 28 out of 29 children eligible for the study participated (Table 2). All children had been fitted with a prosthesis, the mean age of first fitting was 2.8 years (range: 0.5–11 years). In most cases the first prosthesis was cosmetic and was later converted to a myoelectric prosthesis. In 2011, three of the 28 patients had moved abroad; the remaining 25 were invited to revisit the clinic. Two former patients declined participation and six did not respond to the invitation and were thus lost to follow-up. The remaining 17 patients agreed to participate; one of them was unable to visit the clinic due to the travel distance but agreed to answer the questionnaires. These patients' deficiencies were classified according to ISO 8548-1:89, showing that eight were in the upper third deficiency level, five in the middle third, and two in the lower third [26]. One patient had a deficiency at the carpal level. See Table 2 for more detailed information regarding the patients' characteristics.

**Table 2:** Demographics of patients and controls.

	1987		2011	
	Patients	Controls	Patients	Controls
	(n = 28)	(n = 28)	(n = 17)	(n = 34)
Sex [% (n)]				
Male	57.1 (16)	57.1 (16)	52.9 (9)	52.9 (18)
Female	42.9 (12)	42.9 (12)	47.1 (8)	47.1 (16)
Age in years [median (range)]	13 (8–18)	13 (8–18)	37 (32–42)	37 (31–42)
Deficiency (controls: non-dominant side) [% (n)]				
Right	35.7 (10)	17.9 (5)	41.2 (7)	8.8 (3)
Left	64.3 (18)	82.1 (23)	58.8 (10)	91.2 (31)
Prosthesis use* [% (n)]				
Full-time	64.3 (18)	NA	58.8 (10)	NA
Non-full-time	35.7 (10)	NA	41.2 (7)	NA

* Prosthesis use: full-time = more than 8 hours a day, 7 days a week; non-full-time = never to a maximum of 4–8 hours a day, 5–7 days a week.

NA = Not applicable.

### Body structures

#### Structures of the spine

In 1987, the median difference in number of Moiré rings between the deficient and non-deficient side was 1.5 for patients and it was 0.5 between dominant and non-dominant sides for controls, (p<0.001) (Table 3). In 2011, the scoliometer measurements in patients were significantly greater for the thoracic region (p = 0.005), but not for the (thoraco-) lumbar region, compared to their matched controls.

**Table 3:** Body structures of patients and controls.

	1987			2011		
	Patients	Controls	P	Patients	Controls	p
	(n = 28)	(n = 28)		(n = 16)	(n = 32)	
***Structures of the spine***						
Moiré rings difference (median, IQR)	1.5 (1.0–2.0)	0.5 (0.0–1.0)	**<0.001**	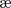	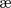	
Thoracic deviations scoliometer (°)	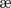	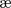		2.2 (2.2)	1,4 (1.8)	**0.005**
Lumbar deviations scoliometer (°)	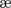	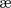		3.1 (1.9)	1.3 (2.0)	0.170
Lumbar deviations perpendicular line (mm)	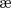	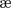		6.6 (6.2)	2.6 (3.3)	**0.025**
***Structures of the trunk*** *						
Scapular size (%)	95.0 (4.1)	100.0 (0.0)	**<0.001**	93.2 (2.4)	100.0 (1.4)	**<0.001**
Thoracic circumference (%)	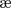	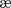		97.2 (1.9)	99.1 (1.7)	**0.001**
***Structures of the arm*** *						
Upper arm length (%)	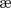	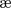		97.0 (2.4)	100.6 (1.8)	**<0.001**
Upper arm volume, total¥(%)	84.8 (8.2)	100.3 (8.2)	**<0.001**	74.6 (12.5)	100.0 (6.6)	**<0.001**
Male	83.8 (7.9)	99.0 (8.7)	**<0.001**	70.5 (13.8)	100.2 (5.5)	**<0.001**
Female	86.2 (8.8)	102.1 (7.9)	**<0.001**	79.9 (8.7)	99.8 (8.0)	**<0.001**
ROM [differences between arms (°)]§						
Shoulder – external rotation	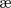	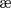		16.3 (14.7)	2.8 (6.5)	**0.002**
Shoulder – abduction	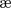	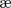		0.4 (7.6)	0.6 (4.2)	0.459
Shoulder – anteflexion	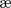	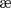		0.6 (9.8)	1.3 (3.1)	0.372
Elbow – flexion	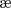	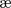		14.9 (13.3)	0.8 (3.3)	**<0.001**
Elbow – extension	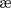	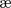		-12.9 (10.6)	0.0 (1.1)	**<0.001**
***Leg length inequality*** (% cases with LLI)	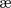	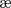		37.5	31.3	0.750

Unless mentioned otherwise, results are described as ‘mean (SD)’. P-values≤0.05 highlighted in bold.

* Scapular size, thoracic circumference, upper arm length and arm volume measures are given for the deficient side as a percentage of the non-deficient side. For the controls, the non-dominant side is measured as a percentage of the dominant side.

§ ROM differences were calculated as the ROM on the non-deficient side minus the ROM on the deficient side.

¥ n = 27 in 1987, both in the patient group and in the control group.

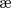
 = Not available.

IQR = Inter Quartile Range, ROM = Range Of Motion.

The perpendicular line measurements gave a mean deviation of 6.6 mm for patients and 2.6 mm for controls (p = 0.025). Ten patients (63%) had a lumbar deviation toward the non-deficient side, whereas two patients had a deviation toward the deficient side.

#### Structures of the trunk

The scapula on the patients' deficient side was, both in 1987 and 2011, smaller compared to their non-deficient side (1987: p<0.001; 2011: p<0.001) and also compared to their matched controls (Table 3). The difference between the patients' two scapulas did not change over time (p = 0.326). The circumference of both thorax halves differed more in patients than in controls (p = 0.001). The mean difference between patients' thorax halves was 1.4±0.8 cm, compared to 0.6±0.5 cm for the controls (p = 0.001). For patients, the smaller thorax half was on the deficient side in all but two cases.

#### Structures of the arm

There was a significant difference in upper arm length discrepancy between patients and controls (Table 3), with the mean difference in upper arm lengths being greater in patients (1.1±0.9 cm versus 0.5±0.4 cm, p = 0.008). The patients' upper arm on the deficient side was shorter compared to the upper arm on the non-deficient side (p = 0.001).

The volume of both upper arms differed more in patients than in controls. The patients' upper arm volume on the deficient side was, both in 1987 and 2011, significantly smaller compared to the upper arm volume on the non-deficient side (1987: p<0.001; 2011: p<0.001). The volume differences between participants' upper arms increased over time (p = 0.008). In contrast, there was no statistically significant difference between the ratio of stump volume and non-deficient forearm over time.

When compared to the controls, the patients' ROM in external rotation of the shoulder and the ROM in flexion of the elbow were restricted on the deficient side, and hyperextension of the elbow was present on the deficient side. All significant differences in the patients' ROM were of clinical relevance (>10°) (Table 3).

Leg length inequality was present in both groups but did not differ significantly (Table 3).

### Physical complaints

When comparing the relative presence of back pain (1987) or physical complaints (2011), there were no statistically significant differences between patients and controls, either in 1987 (14.3% versus 10.7%, p = 0.382), or in 2011 (29.6% versus 20.6%, p = 0.503). In 2011, the patients most often had neck and shoulder pain but none of them had pain in their unaffected hand. In comparison, among controls the pain was mostly located in the back, shoulders, and hand or thumb.

The patients reported a significantly greater disability on the *Quick*DASH compared to the controls (10.4±11.8 versus 2.0±3.3, p<0.001). Furthermore, the scores for the DASH sports/music module differed significantly between patients (n = 11) and controls (n = 24) (14.7±17.0 versus 1.5±6.4, p = 0.001). The deficiency seems to have a minor influence on the patients' (n = 16) ability to work, since no statistically significant difference (5.1±14.4 versus 0.4±1.4, p = 0.139) was found compared to controls (n = 34).

### Body structures and complaints in groups based on prosthesis use

No significant differences between full-time users and non-full-time users of prostheses were found on spinal deviations and physical complaints (Table 4). Furthermore, there was no significant difference in upper arm volume, upper arm length, scapular size or thoracic halves. Neither was there any significant difference between spinal deviation measurements with and without prosthesis.

**Table 4:** Body structures and physical complaints in groups based on prosthesis use.

	Prosthesis use			Intra-patient measurements		
	Full time*	Non-full time*	p	With prosthesis	Without prosthesis	p
***Spinal deviations***						
**1987 (Moiré topography)**	**n = 18**	**n = 10**		**n = 28**		
Difference in contour lines [median (quartile range)]	2.0 (1.0–2.0)	1.0 (0.8–2.0)	0.528	1.0 (1.0–2.8)	1.5 (1.0–2.0)	0.681
**2011 (Scoliometry, perpendicular line)**	**n = 9**	**n = 7**		**n = 13** §		
Thoracic deviations (°)	2.4 (2.0)	3.9 (1.6)	0.148	2.5 (1.8)	3.1 (1.9)	0.194
(Thoraco-) lumbar deviations (°)	1.3 (1.5)	2.9 (2.7)	0.230	1.5 (1.6)	2.0 (2.2)	0.129
Lumbar deviations (mm)	5.7 (5.8)	7.9 (7.0)	0.704	6.1 (5.7)	6.6 (6.2)	0.313
***Physical complaints*** * ***(%)***						
**1987**	**n = 18**	**n = 10**				
Presence of back pain (study-specific questionnaire) (%)	11.1	20.0	0.601	NA	NA	
**2011**	**n = 10**	**n = 7**				
Pain during last week (Brief Pain Inventory) (%)	30.0	28.6	1.000	NA	NA	
DASH-score	9.8 (12.8)	5.4 (8.6)	0.804	NA	NA	

Unless mentioned otherwise, results are described as ‘mean (SD)’. P-values≤0.05 are highlighted in bold.

* Full-time use = prosthesis use at least 8 hours a day, 7 days a week. Non-full-time use = prosthesis use to a maximum of 4–8 hours a day, 5–7 days a week. Prosthesis not fitted during measurements.

§ Three subjects did not have a prosthesis at time of measurements.

NA = Not Applicable.

## Discussion

In this study we have shown that in patients with ULRD, not only the forearm but also the upper arm and trunk are significantly affected by the reduction deficiency. Our study revealed further that spinal deviations were significantly greater in patients compared to matched controls, both in adolescence and in adulthood, though the deviations did not proceed to clinically relevant scoliosis over time. As expected, individuals with ULRD report significantly higher overall disability in the arm, shoulder and hand than their able-bodied peers, with a significant difference in performance of sports and music.

Anomalies in arm and trunk on the deficient side in people with unilateral ULRD have not been described earlier. These anomalies may be the result of under-use of the deficient side and over-use of the corresponding side or another consequence of the insult causing the deficiency in the first place, such as a vascular incident [27], thus affecting the growth of these structures. This is something that needs further research. Irrespective of the cause, the difference in size and ROM may have different impact on the child's future physical health. The results indicate that the side-difference influences the spinal deviations but not with any clinical relevance. In clinical practice, the hyperextension of the elbow is well known by prosthetists from making customized prostheses and probably has a limited future impact. The limited external rotation in the shoulder has been seen in upper-arm clinics but the presence also in below-elbow deficiencies was new to us. It is well known that a visible dysfunction such as ULRD may cause ‘microstressors’ [28] and lead to withdrawn behavior [29], [30]. The limited ROM in the shoulder may be a consequence of avoiding drawing attention to the arm by keeping it still, closely attached to the side of the body. It may also be the result of using the stump only to hold objects in the arm-pit or against a vertical surface. The future consequence of this limited ROM is not known, but the findings may be of clinical importance in the rehabilitation of individuals with unilateral ULRD, as it might show the need for stretching exercises and preventive measures. Further studies and with older people are warranted.

The new findings from this study showing that there is a structural within-person difference between body halves may explain the present findings but also earlier reports of spinal deviations in this group. The moiré topography findings in 1987 indicating that patients with ULRD have a higher susceptibility for deviations of the spine may in fact demonstrate structural anomalies around the scapula rather than rotations in the spine. As such, these deviations did not proceed to clinically relevant scoliosis over time, as shown by scoliometry findings in 2011. Our findings are in agreement with the results of Samuelsson [2], who by using X-rays concluded that patients with ULRD frequently have mild spinal deviations, but of no clinical importance. The spinal deviations seem to be of functional origin and are probably due to postural imbalance due to the asymmetries in arms and trunk. As such, the spinal deviations in ULRD are different from adolescent idiopathic scoliosis. In ULRD, the deviations shown by scoliometry can instead be a result of the within-person difference in truncal circumference. This needs to be studied further. Currently, the spine of a child with ULRD is frequently examined for scoliosis during clinical visits but, according to our findings, this seems unnecessary.

Physical complaints were mostly present in the neck and shoulder and less so in the non-deficient arm, which is in concordance with the results found by Datta [3]. The rates of physical complaints found in this study were remarkably low (29%) compared to other studies, where physical complaints are reported in 40–55% of the cases [3]–[5]. However, these studies all examined patients with upper limb amputations, exclusively or in combination with patients with ULRD. Individuals with ULRD differ from those with upper limb amputations, because they have no sense of loss and will automatically develop compensatory skills for executing the necessary bimanual tasks [31]. Amputees lack these naturally developed compensatory skills; they are used to executing tasks with two hands and they may also have phantom or stump pain prohibiting them from using their stump for execution of tasks [5]. This might be a reason for the difference in physical complaint rates. In contrast, people with ULRD have more years of one-handedness, compared to amputees. As such, more physical complaints in patients with ULRD might be expected. A challenging issue for further research is to answer the question of why there seems to be a difference between physical complaints in patients with ULRD and those with upper limb amputations. Another reason for the difference in physical complaints may be that all our patients have been wearing and using prostheses in various degrees since childhood. This may have had an influence on the development of their body structures, thus influencing the presence of physical complaints. This may also explain why there were no differences due to prosthesis use. Further research is needed with patients with ULRD who have never used a prosthesis, to study the development of body structures under that condition. Furthermore, the patients in our study are relatively young compared to the other studies. In an older sample of patients with ULRD, greater rates of physical complaints may be expected [32]. Finally, all patients in our study have a below-elbow deficiency, while other studies reported on proximal upper limb amputations [3] or both proximal and distal upper limb amputations [4], [5]. The more proximal the amputation, the more likely the person is to suffer problems in the non-affected arm [5].

Individuals with ULRD may choose their work based on their abilities, which may explain why there is no significant difference between the patients' and controls' scores in the DASH work module. Despite their short arm, some of the patients chose bimanual hobbies, such as playing golf and piano, which affected their score on the sports/music module, because they felt they could not perform as well as they wished.

The strength of this study is the long follow-up period of 24 years, which allowed us to present results about patients in their teenage years and as adults. Such a follow-up study has not been presented yet in the international literature. A limitation of the study was the fact that the measurement instrument to assess spinal deviations used in 1987 was unavailable in 2011. As such we were unable to reveal differences in spinal structures over time. The small number of patients in 2011, which decreased the power to detect statistically significant differences, is also a limitation. The number of part-time users and non prosthesis users was small and, thus, it was not possible to make a more precise evaluation of the effects of prosthesis use on body structures and physical complaints. Besides, all patients had been fitted with a prosthesis in their early youth and both full-time wearers and non-full-time wearers varied over time. This means that the influence of prosthesis use on the development of body structures and physical complaints may be evenly distributed within the sample, thus affecting the results on influence of prosthesis use. Further studies with more clear differences between the samples and control for other factors that may cause physical complaints in this population are needed to study this.

## Conclusion

People with unilateral ULRD below the elbow also have structural anomalies of the upper arm, scapula, thoracic circumference and elbow joint on the deficient side. Significant spinal deviations were also found. However, deterioration of the deviations of the spine, probably of functional origin, to clinically relevant scoliosis over time is unlikely. Therefore, regular scoliosis checks for children with ULRD do not seem to be necessary. Further studies are needed on the effect of prosthesis use on the development of body structures and physical complaints in this population.
